# Development of Cost-Effective Fatty Liver Disease Prediction Models in a Chinese Population: Statistical and Machine Learning Approaches

**DOI:** 10.2196/53654

**Published:** 2024-02-16

**Authors:** Liang Zhang, Yueqing Huang, Min Huang, Chun-Hua Zhao, Yan-Jun Zhang, Yi Wang

**Affiliations:** 1 Department of General Practice The Affiliated Suzhou Hospital of Nanjing Medical University Suzhou China; 2 School of Information Science and Engineering Southeast University Nanjing China; 3 The First Clinical Medical College Nanjing Medical University Nanjing China

**Keywords:** NAFLD, artificial intelligence, public health, transient elastography, diagnosis

## Abstract

**Background:**

The increasing prevalence of nonalcoholic fatty liver disease (NAFLD) in China presents a significant public health concern. Traditional ultrasound, commonly used for fatty liver screening, often lacks the ability to accurately quantify steatosis, leading to insufficient follow-up for patients with moderate-to-severe steatosis. Transient elastography (TE) provides a more quantitative diagnosis of steatosis and fibrosis, closely aligning with biopsy results. Moreover, machine learning (ML) technology holds promise for developing more precise diagnostic models for NAFLD using a variety of laboratory indicators.

**Objective:**

This study aims to develop a novel ML-based diagnostic model leveraging TE results for staging hepatic steatosis. The objective was to streamline the model’s input features, creating a cost-effective and user-friendly tool to distinguish patients with NAFLD requiring follow-up. This innovative approach merges TE and ML to enhance diagnostic accuracy and efficiency in NAFLD assessment.

**Methods:**

The study involved a comprehensive analysis of health examination records from Suzhou Municipal Hospital, spanning from March to May 2023. Patient data and questionnaire responses were meticulously inputted into Microsoft Excel 2019, followed by thorough data cleaning and model development using Python 3.7, with libraries scikit-learn and numpy to ensure data accuracy. A cohort comprising 978 residents with complete medical records and TE results was included for analysis. Various classification models, including logistic regression (LR), k-nearest neighbor (KNN), support vector machine (SVM), random forest (RF), light gradient boosting machine (LightGBM), and extreme gradient boosting (XGBoost), were constructed and evaluated based on the area under the receiver operating characteristic curve (AUROC).

**Results:**

Among the 916 patients included in the study, 273 were diagnosed with moderate-to-severe NAFLD. The concordance rate between traditional ultrasound and TE for detecting moderate-to-severe NAFLD was 84.6% (231/273). The AUROC values for the RF, LightGBM, XGBoost, SVM, KNN, and LR models were 0.91, 0.86, 0.83, 0.88, 0.77, and 0.81, respectively. These models achieved accuracy rates of 84%, 81%, 78%, 81%, 76%, and 77%, respectively. Notably, the RF model exhibited the best performance. A simplified RF model was developed with an AUROC of 0.88, featuring 62% sensitivity and 90% specificity. This simplified model used 6 key features: waist circumference, BMI, fasting plasma glucose, uric acid, total bilirubin, and high-sensitivity C-reactive protein. This approach offers a cost-effective and user-friendly tool while streamlining feature acquisition for training purposes.

**Conclusions:**

The study introduces a groundbreaking, cost-effective ML algorithm that leverages health examination data for identifying moderate-to-severe NAFLD. This model has the potential to significantly impact public health by enabling targeted investigations and interventions for NAFLD. By integrating TE and ML technologies, the study showcases innovative approaches to advancing NAFLD diagnostics.

## Introduction

Nonalcoholic fatty liver disease (NAFLD) stands as the foremost chronic liver condition, impacting approximately 1.7 billion individuals worldwide. NAFLD manifests as a multifaceted chronic liver disorder marked by an excessive buildup of fat within liver tissue. As hepatic steatosis advances, it exacerbates the onset and progression of hepatitis and fibrosis, while heightening the likelihood of liver cancer. Furthermore, numerous studies provide compelling evidence linking NAFLD closely with metabolic disorders such as obesity, diabetes, and hypertension, significantly elevating the risk of cardiovascular disease [1]. A recent meta-analysis revealed that the prevalence of NAFLD in Asian countries mirrors that of Western nations. Notably, China exhibits the highest prevalence, incidence, and yearly mortality associated with NAFLD in Asia. If this trend persists, projections suggest that by 2030, the total NAFLD population in China will soar to 314.58 million. Consequently, China will emerge as the global leader in both patients with NAFLD and liver-related fatalities [2]. Given that the majority of NAFLD cases are asymptomatic, early diagnosis holds significant clinical importance. Furthermore, precise quantification of liver fat content and clarification of hepatic steatosis severity are crucial for determining appropriate clinical interventions, assessing disease progression, and evaluating treatment efficacy. Hepatic steatosis is typically categorized into minimal (<5%), mild (5%-33%), moderate (33%-66%), and severe (≥66%) levels [3]. Patients exhibiting moderate-to-severe steatosis necessitate more intensive intervention and follow-up. Enhanced detection of individuals at high risk and early diagnosis can substantially aid in the diagnosis, treatment, and prevention of NAFLD.

Ultrasound is widely acknowledged as the preferred method for screening hepatic steatosis due to its cost-effectiveness, safety, convenience, and efficacy [4]. The characteristic ultrasound findings of fatty liver are either homogeneous or heterogeneous enhancement of liver echogenicity, along with liver enlargement and diminished visualization of intrahepatic ductal structures. However, the accuracy of ultrasound in diagnosing the disease is heavily reliant on the skill and expertise of the operator [5]. Studies have consistently found that ultrasound is highly operator dependent and lacks the capability to precisely determine the extent of hepatic steatosis or distinguish between steatosis and fibrosis, as both conditions lead to heightened liver echogenicity. Consequently, ultrasound is predominantly used for screening fatty liver disease (FLD) [6]. However, it is not recommended for tasks requiring accurate diagnosis and severity grading of early-stage fatty liver, liver transplantation assessments, or evaluation of short-term drug therapies.

At present, liver biopsy stands as the gold standard for diagnosing FLD and evaluating the severity of hepatic steatosis. However, its utility for dynamic monitoring of disease progression and efficacy assessment is limited by factors such as poor patient acceptance and high cost. Additionally, liver biopsy entails inherent risks of complications including invasiveness, bleeding, and infection. Moreover, it is prone to subjective evaluation bias and sampling errors, further impeding its effectiveness as a monitoring tool [7]. Currently, the noninvasive preliminary assessment of hepatic steatosis and the quantitative dynamic evaluation of hepatic fat content represent focal points in current research efforts. Techniques such as transient elastography (TE), computed tomography, and magnetic resonance imaging (MRI) have all been validated for the quantitative diagnosis of steatosis, with MRI demonstrating superior accuracy [6]. However, the high cost, poor patient acceptance, and lengthy examination times associated with biopsy, MRI, and computed tomography render them impractical for large-scale population screening. Consequently, obtaining sufficient sample data in outpatient services worldwide remains challenging. Controlled attenuation parameters in TE represent a quantitative diagnostic approach tailored for detecting steatosis graded as S1, S2, and S3, as well as fibrosis graded as F1, F2, F3, and F4. A meta-analysis has determined that controlled attenuation parameters exhibit good sensitivity and specificity for grading steatosis [7]. Additionally, a prospective study that used TE to assess disease progression in patients with NAFLD indicated that liver stiffness measurements (LSMs) can effectively monitor the degree of liver fibrosis in this patient population [8]. The study indicated that TE can serve as a comprehensive diagnostic tool for both hepatic steatosis and liver fibrosis. It also offers a rapid and noninvasive method to assess liver fibrosis in patients with diverse chronic liver diseases, encompassing chronic hepatitis C, chronic hepatitis B, and NAFLD. Moreover, TE shows promise in predicting complications associated with advanced compensated chronic liver disease [4,9]. However, the widespread adoption of TE in population-based health screenings faces obstacles, particularly in China, where TE is primarily used for evaluating patients with FLD in general hospital settings. There are persistent challenges concerning inadequate ultrasound equipment and insufficient specialized training for physicians in primary health services that require attention. Consequently, there is a pressing need for the development of more cost-effective and efficient methods to identify individuals in the population with FLD who warrant intervention and follow-up, particularly those reaching the threshold of needing medical attention (S≥S2).

Machine learning (ML) offers a promising avenue to tackle these challenges. ML, a branch of computer science, uses algorithms to discern patterns within extensive data sets and predict diverse outcomes [10]. Evolving from pattern recognition and computational learning, ML uses computers to analyze interactions between variables, encompassing both nonlinear and complex relationships, while minimizing errors between predicted and actual outcomes. ML not only enhances predictive accuracy but also has the capacity to identify latent variables that might not be directly observable but can be inferred from other variables. Currently, various ML techniques, including logistic regression (LR), random forest (RF), artificial neural networks (ANNs), k-nearest neighbors (KNNs), support vector machine (SVM), and extreme gradient boosting (XGBoost), are being used in disease prediction with significantly higher accuracy compared with classical methods [11]. Previous studies have used clinical data to diagnose patients with NAFLD, often relying on traditional ultrasound results for FLD diagnosis [12,13]. Given the challenges posed by the unsatisfactory diagnostic accuracy of traditional ultrasound in FLD screening and its inability to provide early warnings for patients requiring follow-up and more stringent interventions, there is an opportunity to leverage data from TE in population-based health examinations. These data can be used to develop a new ML model with enhanced accuracy and the capability to classify patients based on severity thresholds.

## Methods

### Recruitment

All clinical data for our study were sourced from health examinations conducted at 7 health examination centers across Suzhou, encompassing 3 districts of Suzhou Municipal Hospital and its 4 affiliated community hospitals. The study included individuals who underwent health examinations from March to May 2023, with exclusion criteria applied to those lacking TE test results. Among the 1753 patients who underwent TE screening, 1344 were selected during their health examination. Ultimately, a total of 978 health examination records with complete medical files were included, accessible for querying in the case system.

### Ethical Considerations

All participants who agreed to partake in the annual health examination were required to complete an informed consent form. Physical examination data were collected for the Suzhou Municipal Government and Suzhou Municipal Hospital. The authors take full accountability for all aspects of the work, ensuring that any questions regarding the accuracy or integrity of the study are thoroughly investigated and resolved. All procedures adhered to the ethical standards outlined in the Helsinki Declaration and received approval from the Ethical Committee of Suzhou Municipal Hospital. The study was approved by the Ethics Committee of Suzhou Municipal Hospital (ethical approval number K-2022-034-K01).

### Machine and Operational Standard

The machine used in the health examination was the FibroTouch, specifically the Transient Elastography FibroTouch (FibroTouch-FT5000; Wuxi HISKY Medical Technologies). This device assesses the degree of hepatic fibrosis by measuring LSM through vibration-controlled instantaneous elastography. Hepatic steatosis is quantitatively evaluated by measuring the attenuation of ultrasound signals in the liver, known as the ultrasound attenuation parameter (UAP). To address detection errors in patients with obesity, the FibroTouch automatically adjusts the probe based on the thickness of subcutaneous fat following precise positioning and depth measurement. This adjustment ensures comparable diagnostic accuracy to FibroScan [14].

**Figure 1:**
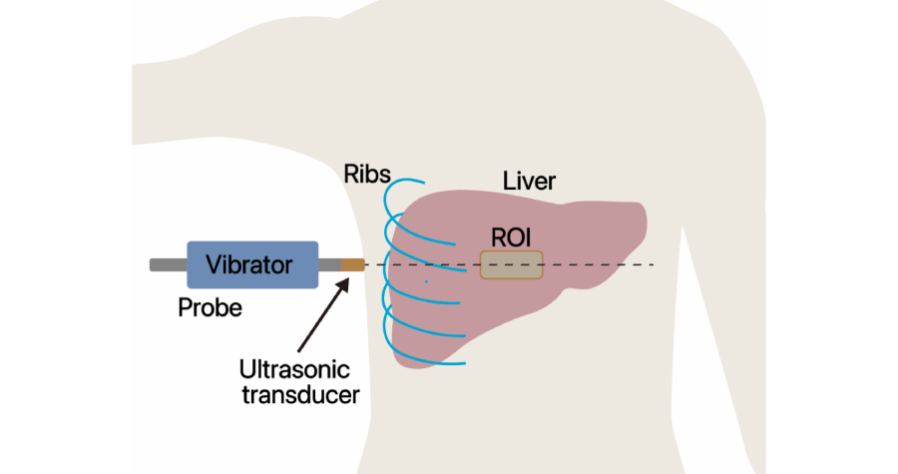
A simplified schematic diagram of TE testing showing an image-guided probe selected to detect the region through the intercostal space. ROI: region of interest; TE: transient elastography.

FibroTouch measurements were conducted by experienced and certified physicians, each having performed over 500 examinations. Following the manufacturer’s instructions, patients assumed a supine position with the right hand placed behind the head to facilitate the expansion of the intercostal space. An image-guided probe was carefully chosen to scan the region between the seventh and ninth intercostal spaces, avoiding cysts and blood vessels in the liver. The probe was maintained in a vertical position relative to the skin surface, with pressure applied within the appropriate range (Figure 1). Detection commenced once the M waveform intensity was uniformly distributed and the A waveform appeared linear. In this study, the representative measurement of FibroTouch was determined by calculating the median value of the 10 acceptable LSMs in kilopascals (kPa) and UAPs in decibels per meter (dB/m), along with their respective IQRs. LSM and UAP measurements were deemed reliable only if 10 successful measurements were obtained, with an IQR-to-median ratio of 30% and a success rate of at least 60% [15].

### Statistical Analysis

**Figure 2:**
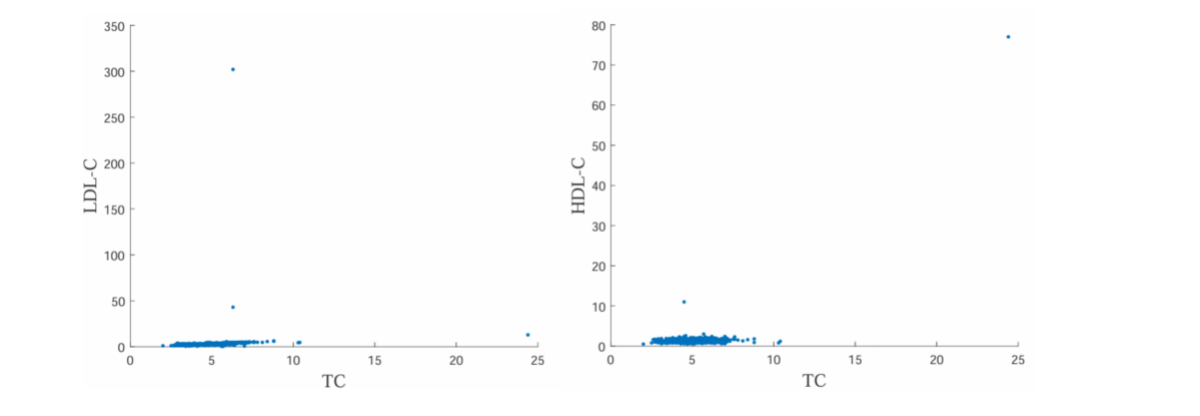
Scatterplots for outlier detection. HDL-C: high-density lipoprotein-cholesterol; LDL-C: low-density lipoprotein-cholesterol; TC: total cholesterol.

When collecting data, we initially searched for the patient’s medical number to ensure that the accessed data did not contain any identifiable patient information. To enhance data quality and mitigate the impact of erroneous data on the model, we developed a comprehensive set of logic algorithms to systematically check for logical errors within the health examination data records. These checks included identifying unit errors, magnitude errors, format errors, and so forth. Any identified errors were then manually corrected following prompts from the algorithm. Additionally, to detect potential hidden errors arising from data entry issues, we generated scatterplots (Figure 2) to identify outliers, which were subsequently monitored and reviewed manually on a case-by-case basis. A professional medical doctor, with over 15 years of experience in the field, assessed the reasonableness of outliers. It is notable that some outliers exhibited values that were theoretically unlikely, such as low-density lipoprotein-cholesterol (LDL-C) levels exceeding 300 mmol/L. We conducted thorough verification of these unreasonable values and subsequently corrected or excluded data entries found to be erroneous due to researchers’ data entry mistakes or other factors.

In the lifestyle characteristics section, dietary information was omitted from the model. This decision was based on the understanding that many older individuals were unable to accurately report their daily food intake, while younger individuals often experienced irregular eating habits. Additionally, in terms of prevalence, conditions with low case numbers such as chronic obstructive pulmonary disease (n=5), myocardial infarction (n=28), and stroke (n=23) were excluded from the model. Osteoporosis was also excluded because bone mineral density assessments were not included in the health examination protocol, making it difficult to ascertain the presence of osteoporosis in the majority of patients undergoing health examinations. Furthermore, 56 patients were excluded due to data disorder errors. Specifically, variables such as red blood cell count, neutrophil count, white blood cell count, and lymphocyte count were mistakenly entered as percentages of red blood cells, neutrophils, white blood cells, and lymphocytes, respectively.

### Characteristic Processing

#### Exercise

The PARs-3 (Physical Activity Rating Scale-3) is a commonly used exercise measurement scale in China [16]. In previous studies, the scale’s internal consistency and reliability were 0.86 and 0.82, respectively. The scale contains 3 dimensions, namely, time, intensity, and frequency of exercise. A score of 20 or higher on this scale is indicative of moderate physical activity, which equates to engaging in at least 150 minutes of moderate exercise per week. To simplify the analysis, moderate physical activity was categorized into 2 groups: individuals who participate in at least 150 minutes of moderate exercise per week versus those who engage in less than 150 minutes weekly.

#### Smoking and Drinking

The health examination records included information on the variety of wine, volume of drinking, and alcohol percentage consumed per day. We calculated alcohol intake using the following equation: alcohol intake (g) = volume of drinking (mL) × alcohol percentage (%, vol/vol) × 0.8 (g/mL). Patients with alcohol intake above the recommended limits (male≥30 g/day; female≥20 g/day) were excluded. However, individuals with an alcohol intake of 0 were included in the analysis. We categorized alcohol intake into 2 groups: those with an alcohol intake of 0 and those with an intake greater than 0, treating them as 2-categorical variables. Similarly, we classified patients into 2 groups based on smoking history: those who had never smoked and those with a history of smoking, also treated as 2-categorical variables.

#### Metabolic Disease

In contrast to previous studies where diastolic and systolic blood pressures were often analyzed as features, we chose not to include them. We believed that the blood pressure measurements of patients undergoing health examinations could be biased due to various factors such as changes in peak blood pressure, medication usage, and clinical hypertension. Similarly, random blood glucose levels are strongly influenced by diet. Instead, we opted to use fasting plasma glucose (FPG), postprandial plasma glucose (PPG), and hemoglobin A_1c_ (HbA_1c_) as variables. Additionally, we included hypertension, hyperuricemia, diabetes, and hyperlipemia as 2-categorical variables. All remaining data were normalized for analysis.

#### Educational Attainment and Financial Situation

Individuals who have never received formal education are classified as “uneducated.” Those with less than a high school education are categorized as “low,” whereas individuals with a high school diploma or specialized education are labeled as “mid.” Education at the college level or higher is defined as “high.” Regarding income, the medical report divides income into categories including below the social minimum wage, slightly below the average social wage, slightly above the average social wage, and significantly above the average wage. Individuals earning below the social minimum wage were categorized as “poor,” whereas those earning significantly above the average wage were classified as “rich.” Those with incomes falling between these extremes were categorized as “average.”

### Enrollment of Participants

**Figure 3:**
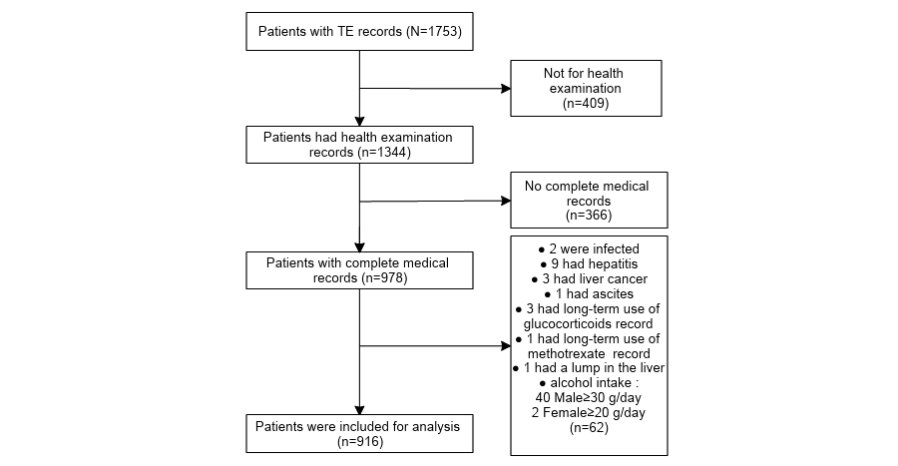
Flowchart of patient enrollment and diagnostic standards of NAFLD from the AASLD Practice Guidance. AASLD: American Association for the Study of Liver Diseases; NAFLD: nonalcoholic fatty liver disease; TE: transient elastography.

We screened individuals who ultimately met the diagnostic criteria for NAFLD based on the American Association for the Study of Liver Diseases (AASLD) Practice Guidance [17]. Cases with conditions known to substantially impact TE results, such as liver cancer and ascites, were excluded from the study. Similarly, individuals with conditions known to affect blood biochemistry analysis, such as infections and long-term glucocorticoid use, were excluded. Figure 3 presents a flowchart illustrating the enrollment process of patients.

All the collated data of the 916 people screened are summarized in Tables 1 and 2. Table 1 summarizes the descriptive characteristics of the study population, including gender, age, educational attainment, financial situation, residence, smoking and drinking state, exercise, and metabolic disease (hypertension, hyperuricemia, diabetes, and hyperlipemia). Table 2 presents a summary of blood biochemistry analysis of the study population.

**Table 1:** Summary of descriptive characteristics of the study population (N=978).

Characteristic				Values, n (%)
**Gender**				
	Male		503 (51.4)	
	Female		475 (48.6)	
**Age (years)**				
	20-60		498 (50.9)	
	≥60		480 (49.1)	
**Finance**				
	Poor		268 (27.4)	
	Average		666 (68.1)	
	Rich		44 (4.5)	
**Education**				
	Uneducated		42 (4.3)	
	Low		262 (26.8)	
	Mid		478 (48.9)	
	High		196 (20.0)	
**Residence**				
	City		911 (93.1)	
	Village		67 (6.9)	
**BMI (kg/m^2^)**				
	<18.5		19 (1.9)	
	18.5-23.9		370 (37.8)	
	24-27.9		430 (44.0)	
	≥28		159 (16.3)	
**Waist circumference (cm)**				
	**Male (n=503)**			
		≥85	338 (67.2)	
		<85	165 (32.8)	
	**Female (n=475)**			
		≥80	299 (62.9)	
		<80	176 (37.1)	
**Smoking**				
	Ever		151 (15.4)	
	Never		827 (84.6)	
**Alcohol (g/day)**				
	**Male (n=503)**			
		0	343 (68.2)	
		0-30	120 (23.9)	
		≥30	40 (8.0)	
	**Female (n=475)**			
		0	453 (95.4)	
		0-20	20 (4.2)	
		≥20	2 (0.4)	
**Sleeping (hours)**				
	<6		154 (15.7)	
	≥6		824 (84.2)	
**Moderate physical activity**				
	No		837 (85.6)	
	Yes		141 (14.4)	
**Metabolic disease**				
	Hypertension		432 (44.2)	
	Hyperuricemia		56 (5.7)	
	Diabetes		258 (26.4)	
	Hyperlipemia		145 (14.8)	

**Table 2:** Summary of blood biochemistry analysis of the study population (N=978).

Characteristic	Values, mean (SD)
Fasting plasma glucose (mmol/L)	6.1 (1.6)
Postprandial plasma glucose (mmol/L)	8.9 (3.2)
Hemoglobin A_1c_ (%)	5.9 (1.2)
Total bilirubin (μmol/L)	13.2 (7.4)
Uric acid (μmol/L)	330.8 (79.1)
Triglyceride (mmol/L)	1.8 (1.4)
Total cholesterol (mmol/L)	4.8 (1.2)
High-density lipoprotein-cholesterol (mmol/L)	1.3 (0.5)
Low-density lipoprotein-cholesterol (mmol/L)	2.9 (0.9)
Alanine transaminase (U/L)	27.9 (16.0)
Aspartate transaminase (U/L)	26.4 (14.5)
γ-Glutamyl transpeptidase (U/L)	36.2 (23.7)
High-sensitivity C-reactive protein (mg/L)	2.2 (3.6)
Platelets (×10^9^/L)	211.8 (54.8)
Hemoglobin (g/L)	136.9 (26.5)
Urea (mmol/L)	8.5 (31.4)
Creatinine (μmol/L)	70.5 (18.5)

### Model Building

To predict NAFLD with different severities, we used several classification models, including LR, KNN, SVM, RF, light gradient boosting machine (LightGBM), and XGBoost.

RF is an ensemble classification algorithm developed by Leo Breiman and Adele Cutler in 1999. It operates by constructing a multitude of decision trees during the training phase and outputs the mode of the classes (classification) or the mean prediction (regression) of the individual trees. RF is widely used for classification, regression, and other tasks. Each decision tree in the RF is built independently by applying the general technique of bootstrap aggregating (bagging), where random samples are selected for the training set. RF determines the final result by aggregating the predictions of all individual trees through a simple majority vote. It has demonstrated high accuracy across various fields, including medical diagnosis. Additionally, RF is frequently used for feature selection in data science workflows. One of the reasons for its popularity in feature selection is due to the tree-based strategies used by RF. These strategies naturally rank features based on how effectively they improve the purity of the nodes in the decision trees. Features that result in the greatest decrease in impurity are typically encountered at the beginning of the trees, while those with the least decrease in impurity are found toward the end of the trees [11]. By selectively pruning trees below a certain node, a subset of the most important features can be derived.

LR is a type of discrete choice model that falls under multivariate analysis. It is extensively used in various fields such as sociology, biostatistics, clinical medicine, quantitative psychology, econometrics, and marketing. LR is often used for empirical analysis and is commonly used for comparison with ML studies [18]. This method offers several advantages, including high power and accuracy, making it a popular choice for modeling binary or categorical outcomes.

ANNs, a family of statistical learning algorithms, draw inspiration from biological neural networks. ANNs have demonstrated remarkable power in nonlinear modeling and have been proven for accurate predictions in many fields, including clinical decision support [19]. The operation of an ANN is akin to a biological neuron, where signals are received through dendrites. In ANNs, this process is replicated with an input layer that feeds into several hidden layers, ultimately leading to an output layer. Each layer consists of numerous perceptrons interconnected by adjustable weights. During training, the ANN iteratively adjusts these weights using a data set, aligning inputs with their desired outputs. This iterative learning process allows the ANN to refine its predictive capabilities over time.

The KNN classification algorithm is among the simplest methods in data mining classification techniques. KNN operates by searching the pattern space for k-training tuples that are nearest to the unknown tuple being classified. These tuples collectively form the KNN classifier for the unknown tuple. The concept of “nearest” is determined by a distance metric, such as the Euclidean distance, which measures the proximity between data points. One potential limitation of KNN classifiers is that they assign equal weight to all attributes based on distance, regardless of their relevance. Consequently, KNN classifiers may suffer from poor accuracy when confronted with noise or irrelevant attributes in the data.

XGBoost is a significantly enhanced implementation of the gradient-boosting supervised ML technique, known for its speed and performance. It shares similarities with RFs but uses a more regularized model formulation to control overfitting. XGBoost operates as a tree ensemble model, which involves the summation of predictions derived from a specific set of classification and regression trees. This regularization technique helps improve the overall performance of the model by mitigating overfitting issues. XGBoost is versatile and can be applied to both classification and regression tasks.

LightGBM is a gradient-boosting framework that uses decision trees as the base learner, similar to XGBoost. However, LightGBM is optimized for efficiency and performance, offering several advantages, including faster training speed and lower memory usage. Additionally, LightGBM supports single-computer multithreading, multicomputer parallel computing, and graphics processing unit training, and has the ability to handle large-scale data.

After cleaning the data, we constructed models (RF and LightGBM) to eliminate irrelevant features such as gender, urea, creatinine, and total cholesterol. The final model incorporated the following features: age, education, finance, alcohol intake, smoking, hypertension, hyperuricemia, diabetes, hyperlipemia, BMI, waist circumference, HbA_1c_, FPG, PPG, total bilirubin (TBil), uric acid (UA), triglyceride (TG), high-density lipoprotein-cholesterol (HDL-C), LDL-C, alanine transaminase (ALT), aspartate transaminase (AST), γ-glutamyl transpeptidase (γ-GT), high-sensitivity C-reactive protein (hs-CRP), platelets, and hemoglobin (HGB).

### Analysis Tools

The basic patient information and paper questionnaire responses were manually entered by a researcher using Microsoft Excel 2019 as the information entry software. Data cleaning, model construction, and area under the curve chart output were performed using Python 3.7 (Python Foundation), with the packages scikit-learn and numpy. The editor used for this purpose was PyCharm (JetBrains). The flowchart was drawn using MyDraw (Nevron Software).

## Results

### Overview of Data Comparison

First, we compared the diagnostic rates of traditional ultrasound with TE and found that traditional ultrasound achieved a high diagnostic rate of 84.6% (231/273) in patients with TE-rated moderate-to-severe steatosis (S≥S2), which is consistent with previous reports on the accuracy of ultrasound diagnosis [20]. The comparison of hepatic steatosis stages produced by TE and traditional ultrasound results is shown in Table 3.

**Table 3:** Comparison of TE^a^ and traditional ultrasound results.

TE	Ultrasound		Total, n (n=916)
	Diagnosed, n (n=502)	Undiagnosed, n (n=414)	
S^b^<S1^c^	81	305	386
S1≤S<S2^d^	190	67	257
S2≤S<S3^e^	133	33	166
S≥S3	98	9	107

^a^TE: transient elastography.

^b^S: hepatic steatosis stage.

^c^S1: mild steatosis.

^d^S2: moderate steatosis.

^e^S3: severe steatosis.

### Model Performance Comparison

Finally, the model incorporated the following features: age, education, finance, alcohol intake, smoking, hypertension, hyperuricemia, diabetes, hyperlipemia, BMI, waist circumference, HbA_1c_, FPG, PPG, TBil, UA, TG, HDL-C, LDL-C, ALT, AST, γ-GT, hs-CRP, platelets, and HGB. The features incorporated into the final model are presented in Table 4. We compared the study population with moderate-to-severe steatosis with those without it.

**Table 4:** Features that were incorporated into the final model and significantly contribute to moderate-to-severe steatosis.

Characteristic	S^a^≥S2^b^	S<S2	*P* value
Age (≥60 years), n (%)	165/273 (60.4)	291/643 (45.3)	<.001
Education (high), n (%)	25/273 (9.2)	163/643 (25.3)	<.001
Physical activity (moderate), n(%)	45/273 (16.5)	87/643 (13.5)	.25
Alcohol (male; 0), n (%)	117/137 (85.4)	224/306 (73.2)	.007
Smoking (male; ever), n (%)	42/137 (30.7)	71/306 (23.2)	.12
Finance (rich), n (%)	7/273 (2.6)	31/643 (4.8)	.12
Hypertension, n (%)	138/273 (50.5)	259/643 (40.3)	.004
Hyperuricemia, n (%)	20/273 (7.3)	29/643 (4.5)	.08
Diabetes, n (%)	88/273 (32.2)	154/643 (24.0)	.009
Hyperlipemia, n (%)	53/273 (19.4)	83/643 (12.9)	.01
BMI (kg/m^2^), mean (SD)	26.0 (2.8)	24.1 (3.2)	<.001
Waist circumference (cm), mean (SD)	90.5 (6.6)	82.6 (7.6)	<.001
Fasting plasma glucose (mmol/L), mean (SD)	6.6 (1.8)	5.8 (1.5)	<.001
Postprandial plasma glucose (mmol/L), mean (SD)	10.1 (4.0)	8.4 (2.5)	<.001
Hemoglobin A_1c_ (%), mean (SD)	6.2 (1.4)	5.9 (1.1)	<.001
Total bilirubin (μmol/L), mean (SD)	14.4 (10.0)	12.4 (5.1)	<.001
Uric acid (μmol/L), mean (SD)	351 (87.0)	317 (71.7)	<.001
Triglyceride (mmol/L), mean (SD)	2.0 (1.6)	1.7 (1.3)	.01
High-density lipoprotein-cholesterol (mmol/L), mean (SD)	1.2 (0.3)	1.3 (0.6)	.03
Low-density lipoprotein-cholesterol (mmol/L), mean (SD)	3.0 (0.9)	2.8 (0.9)	.03
Alanine transaminase (U/L), mean (SD)	29.8 (17.4)	26 (13.6)	.005
Aspartate transaminase (U/L), mean (SD)	27.7 (22.1)	25.4 (9.5)	.02
γ-Glutamyl transpeptidase (U/L), mean (SD)	39.1 (32.8)	33 (16.8)	.001
High-sensitivity C-reactive protein (mg/L), mean (SD)	2.7 (2.9)	1.8 (2.3)	<.001
Platelets (× 10^9^/L), mean (SD)	217 (56.5)	209 (53.9)	.03
Hemoglobin (g/L), mean (SD)	139 (28.6)	134 (26.2)	.02

^a^S: hepatic steatosis stage.

^b^S2: moderate steatosis.

**Figure 4:**
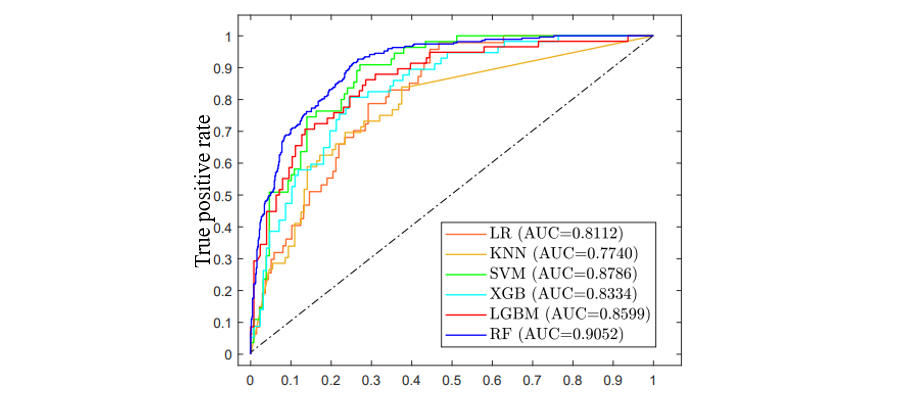
Receiver operating characteristics curve obtained on the test set of the moderate-to-severe fatty liver cohort using the final features. AUC: area under the curve; KNN: k-nearest neighbor; LGBM: light gradient boosting machine; LR: logistic regression; RF: random forest; SVM: support vertical machine; XGBoost: extreme gradient boosting.

Table 5 presents the performance of the classification models. The area under the receiver operating characteristic curve (AUROC) for RF, LightGBM, XGBoost, SVM, KNN, and LR was 0.91, 0.86, 0.83, 0.88, 0.77, and 0.81, respectively. Additionally, the accuracy for RF, LightGBM, XGBoost, SVM, KNN, and LR was 84%, 81%, 78%, 81%, 76%, and 77%, respectively. RF exhibited the best performance. Figure 4 displays the AUROC obtained on the test set of the moderate-to-severe fatty liver cohort using the final features.

**Table 5:** The AUROC^a^, accuracy, sensitivity, and specificity of the 6 classification models.

Model	AUROC	Accuracy	Sensitivity	Specificity
RF^b^	0.91	0.84	0.63	0.92
LightGBM^c^	0.86	0.81	0.63	0.89
XGBoost^d^	0.83	0.78	0.55	0.89
KNN^e^	0.77	0.76	0.60	0.84
SVM^f^	0.88	0.81	0.47	0.95
LR^g^	0.81	0.77	0.52	0.85

^a^AUROC: area under the receiver operating characteristic curve.

^b^RF: random forest.

^c^LightGBM: light gradient boosting machine.

^d^XGBoost: extreme gradient boosting.

^e^KNN: k-nearest neighbor.

^f^SVM: support vertical machine.

^g^LR: logistic regression.

### Model Simplification and Visualization

**Figure 5:**
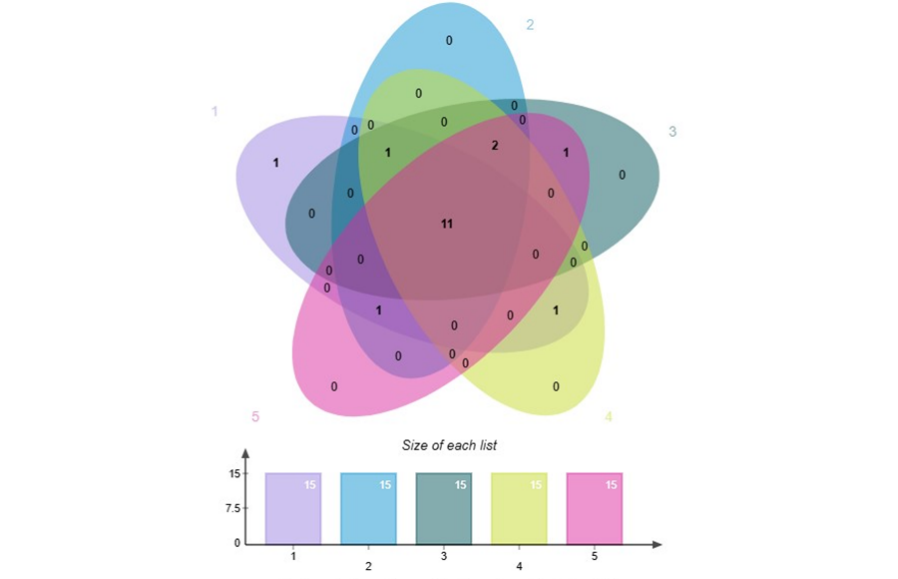
Venn diagram for screening important features.

**Figure 6:**
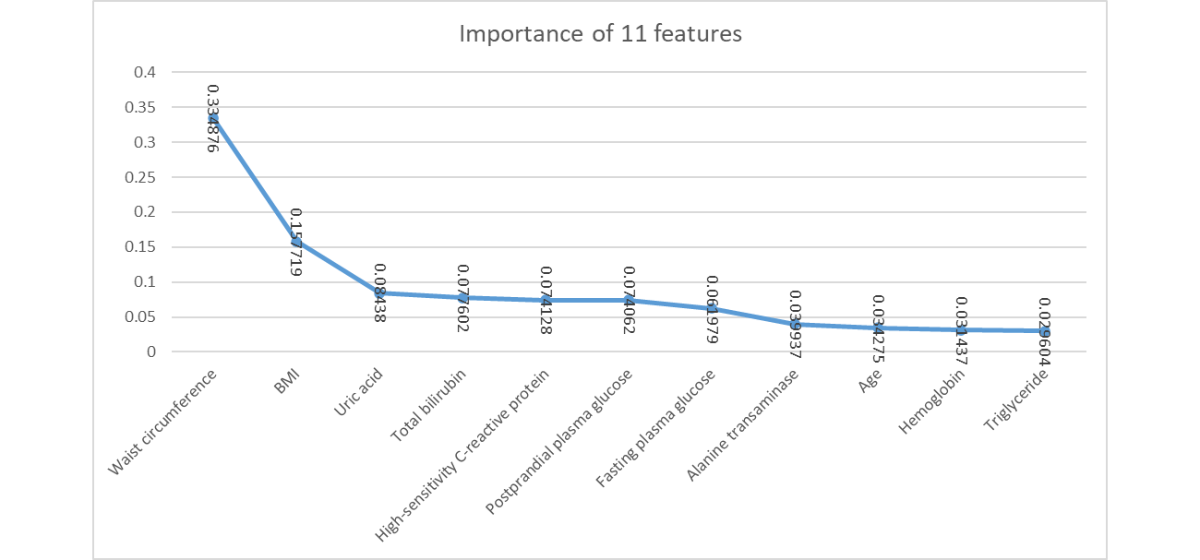
The scree plot, demonstrating the importance of clinical variables obtained through the machine learning modeling on the clinical data.

Furthermore, we attempted to build a more concise model. We repeated the process randomly 5 times, each time selecting the top 15 scored features to create a Venn diagram (Figure 5), resulting in a total of 11 filtered features. We ranked the importance of these 11 features and plotted them on a scree plot (Figure 6). The plot demonstrated a substantial change between FPG and ALT, leading us to choose the first 6 features as inputs, excluding PPG. We made this decision based on the fact that the PPG test takes 2 hours and is not typically performed in most population health examinations.

**Figure 7:**
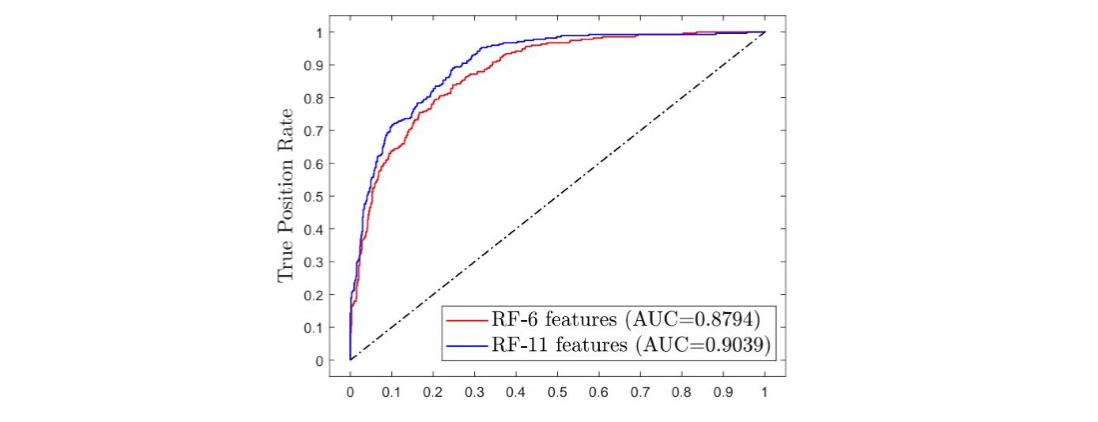
Receiver-operating characteristic curve of simplified RF models. AUC: area under the curve; RF: random forest.

To our surprise, the 6-feature RF model maintained accuracy while simplifying the feature acquisition for training. Table 6 displays the performance of the 11-feature RF model and the 6-feature RF model. The AUROC of the RF (11 features) model is 0.90, with a sensitivity of 0.61 and specificity of 0.94, maintaining the same accuracy as the RF model before simplification. Meanwhile, the performance of the RF (6 features) model showed an acceptable decrease compared with the others, with an AUROC of 0.88, accuracy of 0.82, sensitivity of 0.62, and specificity of 0.90. Figure 7 provides a summary of the ROC curves for the 2 simplified RF models.

**Table 6:** Display of the performance of the 11-feature RF^a^ model and the 6-feature RF model.

Model	AUROC^b^	Accuracy	Sensitivity	Specificity
RF	0.91	0.84	0.63	0.92
RF (11 features)	0.90	0.84	0.61	0.94
RF (6 features)	0.88	0.82	0.62	0.90

^a^RF: random forest.

^b^AUROC: area under the receiver operating characteristic curve.

**Figure 8:**
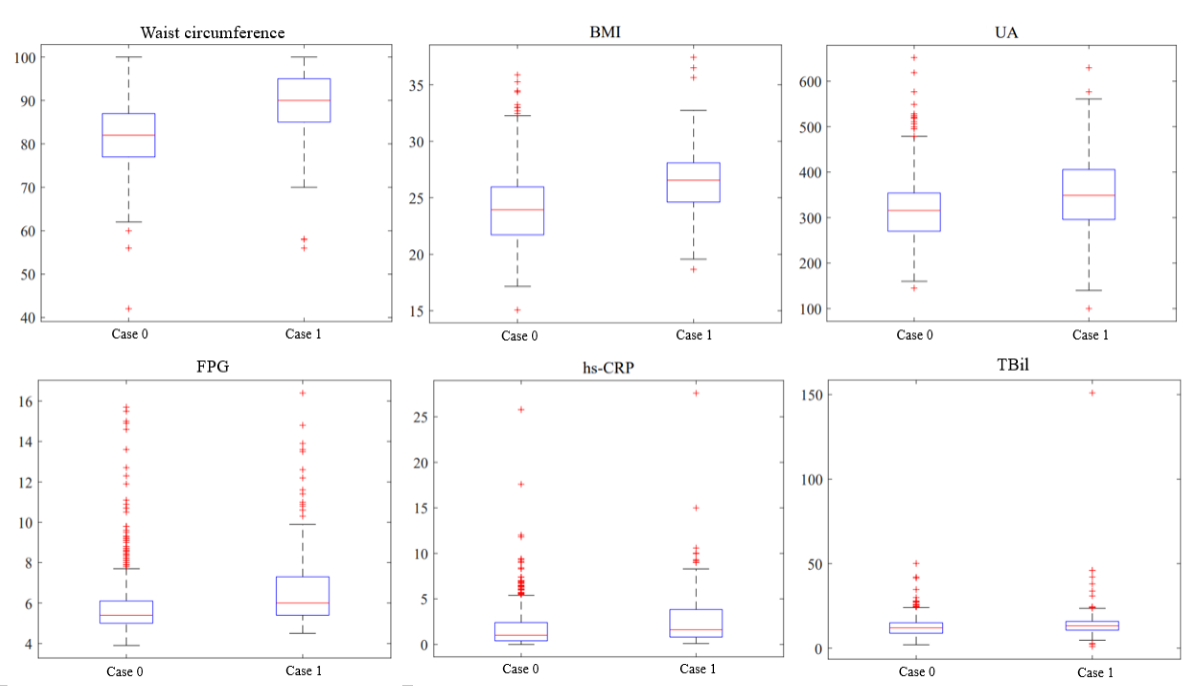
Box diagrams of 6 features in the simplified RF model. FPG: fasting plasma glucose; hs-CRP: high-sensitivity C-reactive protein; RF: random forest; Tbil: total bilirubin; UA: uric acid.

**Figure 9:**
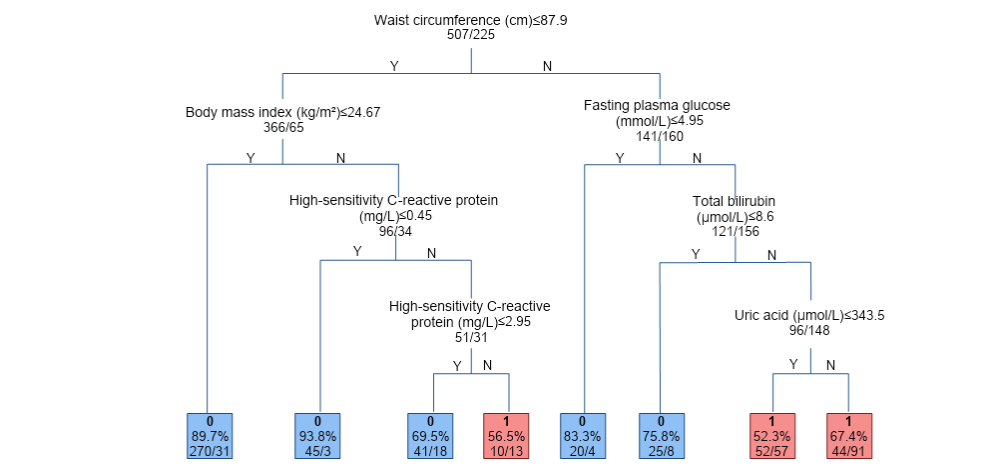
Example tree illustrating some set of rules and thresholds from the dense random forest tree used for classification in the analysis. The “0” and “1” on the leaf node represent moderate-to-severe steatosis and no moderate-to-severe steatosis, respectively; ”Y” means Yes and “N” means no.

Figure 8 presents box plots depicting the distribution of the 6 features. We can clearly see that there is a significant difference (Table 4) in the features between the 2 groups, which again demonstrates the high importance of the 6 features. To present our models more intuitively, Figure 9 showcases an example tree from the dense RF tree used for classification in the analysis.

### User-Friendliness and Cost-Effectiveness

In Suzhou, FPG, UA, and TBil can be tested through blood biochemistry analysis, hs-CRP can be tested through CRP analysis, and BMI and waist circumference are easy to obtain. Our decision tree clearly shows the eigenvalues and prediction accuracy of each node, which is convenient to use in clinical practice. Therefore, we aimed for a simplified decision tree model that can be widely applicable and generalized.

Waist circumference and BMI can be measured during routine physical examinations at a negligible cost. Blood biochemistry analysis costs approximately 168 yuan (US $23.6) per person, while CRP analysis costs around 35 yuan (US $5) per person. The total cost is approximately 203 yuan (US $28.5) per person. As blood biochemistry analysis and CRP analysis are routine checkup items in health examinations, our algorithm does not require additional testing items. Additionally, residents of Suzhou over the age of 40 years have free access to all of these tests once a year, making our model cost-effective.

TE screening, as a noninvasive quantitative assessment of liver fat deposition, poses challenges in determining its feasibility for widespread implementation in densely populated cities such as Suzhou (with >15 million residents), primarily due to the additional expense involved.

## Discussion

### Principal Findings

We used ML to differentiate the severity of NAFLD by incorporating body circumference, lifestyle, and blood indicators. The optimal results demonstrated that a 6-feature RF model could achieve an area under the curve of 88% (with 62% sensitivity and 90% specificity). The 6 features of the RF model were waist circumference, BMI, FPG, UA, TBil, and hs-CRP. The total cost of the indicator tests involved in the RF model was approximately 203 yuan (US $28.5) per person. By contrast, TE, as a noninvasive quantitative assessment of liver fat deposition, currently costs 260 yuan (US $36.5) per person. Compared with the cost of the indicator tests, TE incurs an additional expense of approximately 57 yuan (US $8) per person. Moreover, the Suzhou government offers free annual health examinations to residents over 40 years old within their jurisdiction. Consequently, people in Suzhou, with the assistance of our model, can obtain an almost free evaluation of NAFLD.

Therefore, we ultimately decided to use the RF model composed of these 6 features, as it not only serves as a routine component of health examinations but also has the advantages of being inexpensive and easily obtainable. In addition, we considered that some variables such as systolic blood pressure, diastolic blood pressure, and random blood glucose, which are included in similar studies, may be variable and unreliable. Therefore, we excluded these features to ensure that our model remains stringent and stable.

Metabolic associated FLD is a newer diagnosis; however, NAFLD was still used in this study. This decision was made because in the 43 patients we excluded who met the criteria for alcohol intake (30 g/day for men and 20 g/day for women), the diagnosis of FLD was significantly higher than in the rest of the cohort. Additionally, we considered that the grading data referenced by TE were generated from biopsies of patients with NAFLD, and the inclusion of patients with FLD who exceeded the alcohol intake limit would have led to less rigorous results. In the model, we included 4 metabolic diseases (hypertension, diabetes, hyperuricemia, and dyslipidemia) as 2-categorical variables. Although all 4 diseases showed a correlation with FLD, their importance was deemed lesser compared with features such as waist circumference and BMI.

Models presented in previous studies are typically stratified by age, with age demonstrating a high correlation in the final constructed model. It has been observed that from 2010 to 2018, the annual incidence of NAFLD was higher among those under 60 years of age (4.7%; 95% CI 4.0%-5.5%) than among those over 60 years of age (2.4%; 95% CI 2.1%-2.8%). Additionally, the prevalence of NAFLD is parallel with the rising trend of obesity in China, increasing from approximately 2% in 2000 to 7% in 2014 [21]. Therefore, we believe that the high prevalence across all age groups is highly correlated with changes in lifestyle habits of the population. Consequently, BMI and waist circumference were heavily weighted in our model across all age groups. However, despite their potential significance, lifestyle variables such as smoking, alcohol intake, and physical activity showed low importance compared with the ones we ultimately used, and did not significantly improve the final accuracy. Additionally, a portion of the patients who participated in the TE examination had already been diagnosed with FLD and had undergone lifestyle adjustments.

In economically developed regions such as eastern China, the prevalence of FLD is higher. For example, the prevalence of NAFLD in Shanghai is 38.17% [22]. Ultrasound screening used in population-based health examinations does not allow for a clear diagnosis of FLD grading, and therefore, does not identify patients who need stricter lifestyle control and follow-up. Additionally, there is still a lack of awareness and perception of NAFLD as a chronic disease with serious consequences among the public. Surveys conducted in the 2000s reported that only 31% of the general population in China was aware of NAFLD [23]. If our results are appended to the health examination reports, it may catch people’s attention. Our next plan is to perform TE testing on patients with a model diagnosis of moderate-to-severe NAFLD, and we hope to screen out more patients who need follow-up through the combination of ML and TE examinations. At the same time, TE has the advantage of being less costly and more readily available compared with MRI and biopsy, allowing us to obtain more data to refine our model. We welcome the use of our model for validation. We hope that the use of ML to construct easy-to-use classification models for targeted population screening can be generalized.

### Limitations

Our study has some limitations that should be addressed. First, while our model demonstrated a high specificity, the sensitivity was comparatively lower. This could be attributed to the complexity of our input data, indicating a potential need for higher-dimensional inputs. Second, although we used TE results to classify moderate-to-severe NAFLD along with other categories, it is important to acknowledge that TE itself may not be 100% accurate, necessitating liver biopsy as the gold standard. Incorrect classification could diminish the accuracy of our predictions. It is essential to test the model in real health examinations.

In addition, the clinical data in this study encompassed all age groups above 20 years old. Residents aged 40 years and above can avail themselves of free health examinations provided by the government, wherein related indicators can be included in the examinations to facilitate and reduce the cost of data acquisition. The population composition and dietary habits exhibit good representativeness in the East China region. The relevant research findings also show no obvious preference. However, residents aged 40 years and above generally have more chronic diseases and may be taking medications such as lipid-lowering drugs, which can influence the importance of lipid and other indicators in different age groups. These characteristics may play a significant role in the development of fatty liver, but they have not shown sufficient importance in the application of our model across a broader age range. The accuracy of using these indicators may vary across different age groups. Therefore, if the relevant conclusions of this study are widely promoted, they will require more representative data support to ensure applicability across diverse age demographics.

Finally, it is important to note that while TE offers improved precision and accuracy, studies suggest that obesity increases the risk of TE examination failure [24,25]. Additionally, research indicates that the presence of ascites can lead to failures in ultrasound examinations [25]. These potential failures underscore the need to consider alternative testing strategies when dealing with patients with obesity or ascites, ensuring comprehensive assessment and accurate diagnosis.

### Conclusions

NAFLD has indeed emerged as a significant health burden in China. Unfortunately, many Chinese individuals pay little attention to the disease and are hesitant to undergo expensive tests such as MRI or TE. The proposed cost-effective algorithm using ML to identify moderate-to-severe NAFLD by screening health examination data is promising. This approach has the potential to address the limitations of ultrasound in staging hepatic steatosis and overcome the high cost and low accessibility of TE through the use of artificial intelligence.

## Abbreviations

AASLD: American Association for the Study of Liver Diseases
ALT: alanine transaminase
ANN: artificial neural network
AST: aspartate transaminase
AUROC: area under the receiver operating characteristic curve
FLD: fatty liver disease
FPG: fasting plasma glucose
HbA_1c_: hemoglobin A_1c_
HDL-C: high-density lipoprotein-cholesterol
HGB: hemoglobin
hs-CRP: high-sensitivity C-reactive protein
KNN: k-nearest neighbor
LDL-C: low-density lipoprotein-cholesterol
LightGBM: light gradient boosting machine
LR: logistic regression
LSM: liver stiffness measurement
ML: machine learning
MRI: magnetic resonance imaging
NAFLD: nonalcoholic fatty liver disease
PARs-3: Physical Activity Rating Scale-3
PPG: postprandial plasma glucose
RF: random forest
SVM: support vertical machine
TBil: total bilirubin
TE: transient elastography
TG: triglyceride
UA: uric acid
UAP: ultrasound attenuation parameter
XGBoost: extreme gradient boosting
γ-GT: γ-glutamyl transpeptidase

## Data Availability

The data sets generated and analyzed in this study are available from the corresponding author upon reasonable request.
